# Reply to “Comment on ‘On the Utility of ToxCast™ and ToxPi as Methods for Identifying New Obesogens’”

**DOI:** 10.1289/EHP1122

**Published:** 2017-01-01

**Authors:** Amanda S. Janesick, Giorgio Dimastrogiovanni, Raquel Chamorro-Garcia, Bruce Blumberg

**Affiliations:** 1 Department of Otolaryngology–Head & Neck Surgery, Stanford University School of Medicine, Stanford, California, USA; 2 Department of Developmental and Cell Biology, University of California, Irvine, Irvine, California, USA; 3 Department of Environmental Chemistry, IIQAB-CSIC (Superior Council of Scientific Investigations), Barcelona, Spain; 4 Department of Pharmaceutical Sciences, University of California, Irvine, Irvine, California, USA

We thank Houck et al. for taking the time to critique our paper, which evaluated the utility of ToxCast™ bioactivity data and the ToxPi tool for predicting PPARγ activation and induction of adipogenesis. Our science-based critique of the performance of certain ToxCast™ assays should have elicited a response that engaged with the substance of our paper. Instead, Houck et al. highlight irrelevant or marginally relevant points and criticize us for analyses performed by scientists at the U.S. Environmental Protection Agency (EPA) while ignoring what we consider to be fundamental problems with ToxCast™ assays. Here we respond to their major points.

In their first point Houck et al. suggest that we failed to cite the correct publications for the NovaScreen®, GeneBLAzer®, Attagene, and Tox21 assays. We cited the original developers of these assays and Knudsen et al. (2011) for using them in ToxCast™ Phase I to profile 309 chemicals. We did not perform targeted testing of Tox21 assays.

In their second point Houck et al. make multiple criticisms regarding the assays, our analysis of them, and possible reagent differences. We want to clarify that we undertook this project as a collaboration with Dr. Kristina Thayer of the National Toxicology Program (NTP) in 2010 as an activity related to the January 2011 NTP workshop “Role of Environmental Chemicals in Diabetes and Obesity” (Thayer et al. 2012). Prior to the workshop, Dr. Thayer provided us with a list of PPARγ activators generated by EPA scientists during late 2010. We agreed to test the top 20 ranked chemicals if they were provided.

In rechecking our material transfer agreements, we found that the top 20 PPARγ hits were indeed provided by the EPA at the request of the NTP; it was our understanding that these were from the same stocks as were used in ToxCast™. We added an additional chemical, chlorothalonil (purchased separately), because the ToxCast™ Novascreen^®^ assay results suggested it bound avidly to PPARγ (AC_50_ = 0.54 μM). Following the workshop, we were provided with a second group of compounds (the “ToxPi chemicals”) by the NTP (material transfer agreement dated 27 October 2011). ToxPi chemicals were generated by prediction models based on assays applicable to biological processes associated with diabetes and obesity, assays that were nominated by experts in obesity, diabetes, and metabolism. The adipocyte differentiation model, which my group helped construct, was based on assay results for PPARγ, PPRE, RXR, GR, LXR, LXRE, SREBP1, and C/EBP; see Table S2 in Auerbach et al. (2016) and Figure 3 in our paper. Prediction models were generated by Dr. David Reif, then of the EPA, and the results provided to the investigators by the NTP, together with test chemicals. These models reflected the data analysis pipeline used by the EPA in 2011. It was our understanding that these chemicals were from ToxCast™ stocks, but whether this was the case is unclear in our correspondence. We tested all of the chemicals provided to us in good faith and reported the results.

Changes in the EPA data processing pipeline caused the lists of prioritized chemicals to change numerous times between 2011 and 2015. Auerbach et al. (2016) used a data processing pipeline that evolved during the period of 2014–2015. Their prediction models utilized ToxCast™ Phase II data on 1,860 compounds; the original 2011 ToxPi prioritized chemicals were based on 309 ToxCast™ Phase I chemicals. Therefore, the targeted testing analysis originally contemplated and undertaken by multiple groups was no longer straightforward, and Auerbach et al. (2016) was published as a review article.

Surprisingly, none of the chemicals on the 2011 list that we identified as active on PPARγ, on RXR, and in adipogenesis assays were included on the 2015 list of prioritized chemicals (see Table 2 in Auerbach et al. 2016). Three chemicals appeared on both lists (tebufenpyrad, pyridaben, and fenpyroximate) but did not induce adipogenesis. That none of the active chemicals from the 2011 ToxPi list appeared in the 2015 list, whereas 3 inactive chemicals were incorrectly predicted to be active on both lists, indicates that the prioritization process needs improving. It is instructive to consider where the chemicals we found to be active are ranked by Auerbach et al. (2016). The 1,890 ToxCast Phase II chemicals were ranked from 1 to 810 by EPA scientists using the 2011 ToxPi list of assays; position 810 comprised 1,050 chemicals with a score of 0 (See Table S3 in Auerbach et al. 2016). The chemicals active in our assays were ranked as follows: tebupirimfos, 48; triphenyltin hydroxide, 71; spirodiclofen, 96; triflumizole, 150; zoxamide, 223; bisphenol A, 290; quinoxyfen, 444; flusilazole, 490; fludioxonil, 525; forchlorfenuron, 663; and pymetrozine and acetamiprid, 810. Therefore, the source of the low ranking is the ToxCast™ assays themselves, not the source of the chemicals. Since both the 2011 and the 2015 lists were generated by EPA scientists, it is unclear how our analysis of which chemicals to test was faulty, as Houck et al. allege.

Houck et al. state that we did not consider chemical source in our discussion of why the assay results in our study disagreed with ToxCast™ results. While it is possible that one batch of a chemical has a contaminant that produces spurious activity, or has degraded such that the active material is no longer active, we minimized this possibility by receiving test chemicals from reputable sources (the NTP and the EPA). These chemicals were used exclusively for receptor activation assays and in most of the adipogenesis assays. In some cases, the stock of chemicals provided to us was exhausted, and we repurchased them from commercial sources. We did not observe differences in the ability of the repurchased chemicals to induce adipogenesis from the originals provided by the NTP and the EPA.

Houck et al. further state that we did not consider methodological and platform differences in our criticism of the results of ToxCast™ assays. We have been doing nuclear receptor activation assays since 1992 and have contributed to the development of these technologies and the interpretation of results. While it can be made to appear that there are differences in our techniques that would obviate comparison of receptor activation assays across species or platforms, this is not the case for PPARγ and virtually all other nuclear receptors (with the exception of the xenobiotic receptors SXR/PXR and CAR, which exhibit strong species selectivity for a subset of compounds). Occasional differences arise in PPARγ activation across species, but these are exceptional. It is also possible that a chemical can act as a receptor agonist in one cell type and be inactive or a receptor antagonist in another. Such chemicals are uncommon, and one would not expect to find many, if any, among the 37 PPARγ or PPRE activators identified by the ToxCast™ assays we tested.

Whether chemicals can be metabolized to active forms by the cells used in the Attagene assays is also unlikely to be a valid criticism. The ability of chemicals to cause hits in the NRF2 assays noted by Houck et al. (information that was not conveyed to us when we received these chemicals to test) and the disproportionately high number of hits generated by the Attagene *trans*-factorial assays indicates that these assays are prone to false positives when the cells are subjected to oxidative stress, not an indication of metabolism to receptor activators. Indeed, 143 out of 309 ToxCast™ Phase I chemicals activated PPARγ in the ToxCast™ Phase I Attagene PPARγ assay results. In late 2010 our interest and experience in identifying potential new obesogens prompted us to test whether these assay results were reproducible. The large disagreement among the Attagene PPARγ, Novascreen^®^ ligand binding, and GeneBLAzer^®^ assays we noted should raise serious doubts about the accuracy of all these assays (see Figure 8 in our paper). Instead, Houck et al. suggest that we erred by identifying discrepancies between ToxCast™/Tox21 PPARγ assays and our assays, while they ignore the discrepancies among ToxCast™ and Tox21 assays.

In their third point Houck et al. suggest that our adipogenesis and gene expression assays do not completely agree with each other for all chemicals at every dose. We note that these assays are independent from each other, evaluating different end points. An adipogenesis assay measures the accumulation of neutral lipids in cells. It is immaterial that some chemicals induced lipid accumulation without inducing expression of every adipocyte marker gene. The data suggest that these cells may be partially differentiated intermediates rather than fully differentiated adipocytes. It is well known that chemicals might have activity at one concentration in a receptor activation assay and at a different concentration in an assay that measures a different end point, such as lipid accumulation; the former is not a prerequisite for the latter. ToxCast™ Phase I did not measure adipogenesis, thus, we had nothing to compare with our adipogenesis assay data.

In their fourth point Houck et al. state that we made erroneous conclusions regarding the relative selectivity of the RXR-active chemicals. This point deserves discussion because it gets to the heart of what we believe is lacking in ToxCast™—expert interpretation of the data analysis that the EPA makes freely available to the public. We correctly noted that it is biologically implausible that the ToxCast™ RXR assays show a large number of subtype-selective chemicals. Houck et al. state that their results generated with a chimeric GAL4-DNA binding domain/RXRβ ligand binding domain are reproducible (i.e., precise). However, well-performed assays should be accurate (reflecting reality) as well as precise.

Strong evidence that ToxCast™ PPARγ and RXR assays are problematic is evident when comparing results from ToxCast™ Phase I and Phase II (see Figure S8 in our paper). When the same contractors tested the same chemicals in the same assays in Phase II as they had tested in Phase I, the results were highly discordant (*R* ^2^ of 0.42 for the ATG GAL-PPARγ assay and *R* ^2^ of 0.18 for the NovaScreenÆ PPARγ binding assay). Moreover, there is little agreement with the results of ToxCast™ and Tox21 assays on the same targets (see Figure 8 of our paper). We showed that results of Attagene PPARγ activation assays overlapped more closely with Tox21 PPARγ antagonist assays than with Tox21 PPARγ activation assays (see Figure S8 in our paper). Notably, Judson et al. (2015) show the same close correspondence between Attagene ERα activation and Tox21 ERα antagonist assays (see the left 4 columns of Figure 2 in Judson et al. 2015), suggesting to us there is a problem with Attagene or Tox21 assays rather than an error of interpretation on our part.

Houck et al. go on to question our statement about triphenyltin being a false negative. We are aware that triphenyltin is a PPARγ and RXR activator (see Grün et al. 2006) and noted that the chemical we were provided, fentin, was not listed as an RXRα activator the ToxCast™ data used to generate the 2011 ToxPis. This is a trivial point because we (Grün et al. 2006) and others (Kanayama et al. 2005) showed that triphenyltin is a potent activator of RXRs and PPARγ despite its cytotoxicity,

Houck et al. further state *1*) that we erred in selecting chemicals active only in the ATG PPARγ *trans*-factorial assay and not the ATG PPRE assay, *2*) that we did not use proper statistical considerations, *3*) that we apparently considered only potency and ignored efficacy, and *4*) that we misapplied the *Z*-score metric. As noted above, these chemicals were prioritized by EPA scientists from a ToxPi they generated based on assay end points selected by experts at the 2011 NTP obesity and diabetes workshop. Thus, any statistical or methodological errors in the generation of the prioritized list of chemicals for targeted testing were made by EPA scientists, not by us. The same assays were used for the ToxPis by Auerbach et al. (also generated by EPA scientists) and produced a very different prioritized chemical list (Auerbach et al. 2016). The assays utilized to generate both ToxPis included the ATG PPRE assay (see Figure 3 in our paper). It is unclear whether we misapplied the *Z*-score metric in our analysis of ToxPis generated from ToxCast™ Phase II assay results because *1*) Houck et al. do not state what error(s) we may have made, *2*) we used *Z*-scores calculated by EPA scientists available on the ToxCast™ dashboard (https://www.epa.gov/chemical-research/toxcast-dashboard), *3*) we applied the same equation that weighted AC_50_ values with *Z*-scores developed by EPA scientists (Auerbach et al. 2016), and *4*) we did not have access to the publication they cite (Judson et al. 2015) because it appeared online on 13 August 2015, nearly 2 months after the submission of our paper on 18 June 2015.

Our point about *Z*-score corrections eliminating true positives still stands. A prime example is fludioxonil, which we showed to be an RXRα activator that promoted adipogenesis in mesenchymal stem cells and 3T3-L1 preadipocytes (see Figures 4–6 in our paper). Houck et al. mistakenly state that we reported fludioxonil to be inactive, and therefore, we should not be surprised at its low *Z*-score. Given its low *Z*-score, our ToxPi recalculations demonstrated that fludioxonil would have been ranked very low as verified by Auerbach et al. (2016) where fludioxonil was ranked 525 out of 810 by EPA scientists.

In their Table 1, Houck et al. confuse PPARγ activation with our ToxPi and adipogenesis data. We incorporated *Z*-scores into our recalculated ToxPis (see Figure S5A in our paper) following the methods of Auerbach et al. (2016). These ToxPis included more assays and *Z*-scores than solely PPARγ (e.g., RXR, GR, LXR, SREBP, CEBP). Hence, the text they quote from our paper about “true positives” was referring to the ToxPi data (a collection of numerous assays), but their critique and Table 1 incorrectly refer only to PPARγ activation.

It is concerning when ToxCast™ assays evaluating the same end point are not congruent (see Figure 8 in our paper). Instead Houck et al. state that no single ToxCast™ assay can be taken as truth and attempt to apply various computer models to identify assay positives. In our view, it is unlikely that post hoc computational analysis will compensate adequately for poor-quality assays. Instead, the poorly performing assays need to be corrected or eliminated in favor of assays that show the expected degree of congruency when the same end points are evaluated. Well-performed receptor activation assays should be highly accurate and reproducible within a laboratory and across laboratories.

In their final point, Houck et al. suggest that they do not average results across assays as a matter of standard practice. In fact, this is precisely what the ToxPi tool does, as clarified by Reif et al. (2010). Averaging the results of highly congruent assays on the same end point might be useful but is problematic when the assays are not congruent. This is a weakness of the ToxPi tool—it averages the results of ToxCast™ assays (that may not perform equivalently) to prioritize chemicals for further analysis. We correctly pointed out this shortcoming. We are aware that EPA scientists have developed a series of complex computational network models to predict estrogenicity of chemicals based on ToxCast™ assays (see for example Judson et al. 2015 and Rotroff et al. 2014). Unfortunately, these models produce discordant results (see Appendix 4 of Supplemental File S1 in Judson et al. 2015) whereas ligand binding, co-activator recruitment, and receptor activation assays on estrogen receptor alpha should be highly congruent, as should ligand binding and receptor antagonist assays. Ongoing changes in the evolving data processing pipeline become problematic for targeted testing that is planned and implemented ahead of those changes, as was the case with the 2011 NTP obesity and diabetes workshop that ultimately led to the review article by Auerbach et al. (2016) (K. Thayer, personal communication). Further ongoing changes are also evident in the EPA models for estrogenicity noted above. To help resolve issues discussed here, we recommend independent targeted testing of these various models and lists of prioritized chemicals by experts in the field to aid in the refinement and calibration of the models.

We appreciate the free availability of ToxCast™ and Tox21 assay results. This sort of testing is inarguably the future of “21st century toxicology.” The scientific literature abounds with publications touting the predicted utility of ToxCast™ (121 citations as of 15 September 2016) and Tox21 (66 citations as of 15 September 2016). However, there is a paucity of targeted testing and secondary screening to verify, refine, and calibrate the results of ToxCast™ and Tox21 assays—these will be indispensable to maximize the utility of these important programs.
